# Perfluorocarbon Emulsion Contrast Agents: A Mini Review

**DOI:** 10.3389/fchem.2021.810029

**Published:** 2022-01-10

**Authors:** Ryan Holman, Orane Lorton, Pauline C. Guillemin, Stéphane Desgranges, Christiane Contino-Pépin, Rares Salomir

**Affiliations:** ^1^ Image Guided Interventions Laboratory (GR-949), Faculty of Medicine, University of Geneva, Geneva, Switzerland; ^2^ University of Avignon, CBSA-IBMM, (UMR5247), Avignon, France; ^3^ Radiology Department, University Hospitals of Geneva, Geneva, Switzerland

**Keywords:** contrast agents, emulsions, perfluorocarbons, fluorine chemistry, radiology

## Abstract

Perfluorocarbon emulsions offer a variety of applications in medical imaging. The substances can be useful for most radiological imaging modalities; including, magnetic resonance imaging, ultrasonography, computed tomography, and positron emission tomography. Recently, the substance has gained much interest for theranostics, with both imaging and therapeutic potential. As MRI sequences improve and more widespread access to ^19^F-MRI coils become available, perfluorocarbon emulsions have great potential for new commercial imaging agents, due to high fluorine content and previous regulatory approval as antihypoxants and blood substitutes. This mini review aims to discuss the chemistry and physics of these contrast agents, in addition to highlighting some of the past, recent, and potential applications.

## Introduction

Typical radiological contrast agents are generally iodinated substances for computed tomography (CT) and are gadolinium-based substances for magnetic resonance imaging (MRI). Liquid perfluorocarbon emulsions have been well studied as a diagnostic contrast agent, but has not received regulatory approval for routine clinical use as an intravenous contrast agent by the United States Food and Drug Administration (FDA) or the European Medicines Agency (EMA).

Liquid and gaseous perfluorocarbons have been used in commercial imaging agents. The phase of the perfluorocarbon at physiological conditions is generally dependent on the boiling point, which varies with the molecular weight of the substance; though some substances, like perfluorooctylbromide (PFOB), do not completely adhere to this trend due to intermolecular interactions (Cosco et al., 2015). The optimal perfluorocarbon formula varies with the application. For instance, ultrasonography implements gaseous perfluorocarbon in microbubbles as contrast agents; while, ^19^F-MRI uses unemulsified gases and high density perfluorocarbon emulsions to increase fluorine signal (Cosco et al., 2015).

Due to the high oxygen solubility, perfluorocarbons were heavily studied as antihypoxants and blood substitutes. Later generations benefited from a reduced side-effect profile, longer storage life, ability to be frozen, ability to be sterilized by autoclave, a more uniform size distribution, and shorter accumulation times in tissue (Vorob’ev, 2009). Many alternative formulas and experimental applications, like radiological contrast agents, have developed alongside. Methods like X-ray CT and MRI allow quantitative concentration measurements and improved tissue contrast (Mattrey et al., 1990; Riess, 2001). Many recent preclinical studies of potential liquid perfluorocarbon emulsions are aimed at theranostic (*i.e.,* therapy and diagnostic) capabilities and alternative applications. The clinical and preclinical studies include drug delivery (Al Rifai et al., 2020), adjuvants for blood-brain barrier opening (Peng et al., 2018), anti-ischemics and antihypoxants (Leese et al., 2000; Noveck et al., 2000; Hill et al., 2002; Kachalina et al., 2007; Kligunenko et al., 2007; Moroz et al., 2007; Yermolenko et al., 2007; Culp et al., 2019; Huang et al., 2020), liquid ventilation therapy (EU/3/20/2383, 2021; EU/3/20/2361, 2021), radiological contrast agents (Mattrey et al., 1987a; Mattrey et al., 1987b; Bruneton et al., 1988; Mattrey et al., 1988; Mattrey, 1989), and thermal enhancement for focused ultrasound ablation (Zhang et al., 2011; Desgranges et al., 2019; Lorton et al., 2020).

## Synthesis and Physiochemical Properties of Perfluorocarbon Contrast Agents

Perfluorocarbons can be generated from hydrocarbons by fluorination to substitute the hydrogen atoms with fluorine, with PFOB being of great interest for imaging agents (Riess, 2001). Of primary commercial benefit to the use of PFOB is the capability for large-scale production. The molecule can be derived in a high yield, by a one-step telomerization process through direct bromination of the F-alkyl iodides used in the production of *Teflon* (Riess, 2001). As perfluorocarbons are immiscible in aqueous solution, these require emulsification for stability (Corvis et al., 2018). The emulsifiers are often phospholipids, poloxamers, or fluorosurfactants. Phospholipids are based on egg lecithin isolated from egg yolk through solvent extraction, and composed largely of phosphatidylcholine (Gobley, 1846; Hensing, 2004). Poloxamer surfactants include *Pluronic-F68* used as an emulsifier in *Fluosol* emulsions and *Proxanol-268* used as an emulsifier in *Perftoran* (Riess, 2001). Poloxamers are made with high polydispersity for other industrial applications, and can be purified for medical grade applications (Riess, 2001). Amphiphilic fluorosurfactants are composed of a fluorinated tail group and a hydrophilic head group, and allow very low interfacial tension (Riess, 2001). The lethal doses (LD_50_) of some selected poloxamers and fluorosurfactants are *Proxanol-268* at 20 g. kg^−1^, *Pluronic-F68* at 9.4 g. kg^−1^, and F-TAC at 4.5 g. kg^−1^ in rodents (Vorob’ev, 2009; Maurizis et al., 1994).

The industrial process of large-scale emulsions manufacturing is well developed in pharmaceutics and has long been implemented in the production of parenteral nutrition (Riess, 2001). The emulsion solutions have generally been produced through sonochemical ultrasonic processes which are linked with cavitation, where cavitation nuclei originate from small air bubbles or dust particles in solution, imploding upon excitation to promote further emulsification (Canselier et al., 2002). Cavitation events form when the fluid hydrodynamic pressure becomes lessened to the vapour pressure (Bondy and Söllner, 1935). The vapour pressure of perfluorocarbons in emulsion contrast agents (about 1–3 kPa) are comparable, but slightly lower than water (6.3 kPa) and blood plasma (6.4 kPa) at physiological temperature (Vorob’ev, 2009; Grollman, 1928). These effects will alter the emulsification process, including the droplet diameter and size distribution. Short sonication times tend to generate larger droplets while longer sonication times result in smaller droplet size (Canselier et al., 2002). Cavitation effects can be enhanced with lower ultrasound frequency, lower acoustic pressure, lower medium viscosity, lower medium surface tension, higher energy density, and higher acoustic intensity (Lorimer and Mason, 1987; Canselier et al., 2002). Cavitation implosion effects are also reduced in solvents with higher vapour pressures or at increased temperatures that raise vapour pressure in the fluid-vapour mixture in cavitation sites (Canselier et al., 2002).

Early investigations noted that the incorporation of chlorine or bromine atoms into the perfluorocarbons resulted in faster excretion rates, not predicted based on molecular weights alone (Kabalnov et al., 1992; Riess, 2001). This halogen gives the molecule lipophilic character and enhances clearance rates by allowing the molecule to bind to circulating lipids *en route* to pulmonary excretion (Long et al., 1972a; Long et al., 1982a; Kabalnov et al., 1992; Weers, 1993; Riess, 2001). Although perfluorocarbons have low hydrocarbon affinity, halogen bonding is observed in some systems, to generate self-assembly of supramolecular and crystalline structures (Fox et al., 2004). In these systems, there is a non-covalent interaction between a halogen atom in a perfluorocarbon molecule that acts as an Lewis acid electron acceptor and an atom that acts as a Lewis base electron donor. Similar to hydrogen bonding effects, the halogen atoms are prone to accept electron density from the free electron pairs in neighboring molecules, as the fluorine atoms have a strong electron withdrawing effect (Fox et al., 2004).

The high gas solubility in the liquid perfluorocarbons can be explained by the nonpolar nature of both molecular species. The perfluorocarbons show low polarity and low polarizability, allowing the molecules to readily dissolve molecular gases like noble gases, oxygen, nitrogen, and carbon dioxide (Riess, 2001; Dias et al., 2004). The low polarizability generates a lipophobic character while the overall nonpolar character leads to hydrophobicity (Riess, 2001; Israelachvili, 2015). Some physical and chemical properties of PFOB are given in Table 1.

**TABLE 1:** PFOB Physical Properties

Molecular formula Riess, (2001)	C_8_F_17_Br
Molecular weight, (g.mol^−1^) Astafyeva et al. (2015)	500.0
Molar Volume, (Å^3^) Riess, (2001)	432.0
Boiling point, (°C) Astafyeva et al. (2015)	143.0
Melting point, (°C) Riess, (2001)	5.0
Critical solution temperature (n-hexane, °C)	-20.0
Solubility in water, (mol.m^−3^) Riess (2001); Astafyeva et al. (2015)	5.0E-6
Ostwald coefficient for O_2_ in PFOB, 308 K Deschamps et al. (2007)	0.5
Ostwald coefficient for CO_2_ in PFOB, 308 K Deschamps et al. (2007)	2.2
Henry’s constant for O_2_ in PFOB, (MPa at 308 K) Deschamps et al. (2007)	21.6
Diffusion coefficient, m.s^(−1)^ Astafyeva et al. (2015)	5.2E-10
Density, (kg.m^−3^) Astafyeva et al. (2015)	1920.0
Sound velocity, (m.s^−1^) Astafyeva et al. (2015)	631.8
Refractive index (at 298 K) Riess, (2001)	1.3
Adiabatic compressibility, (m.kg^−3^.Pa^−1^) Astafyeva et al. (2015)	6.9E-13
Surface tension, (mN.m^−1^) Riess, (2001)	18.0
Interfacial tension with water, (mN.m^−1^) Astafyeva et al. (2015)	48.7
Spreading coefficient, (mN.m^−1^) Riess, (2001)	2.7
Vapour pressure, (kPa at 37 °C); Dimitrov et al. (2016)	1.3
Heat of vaporization, (kJ.mol^−1^) Riess, (2001)	4.8
LD_50_ in rodents, (g.kg^−1^) Kim et al. (2021)	14.7
Toxic hazard classification by Cramer Dimitrov et al. (2016)	High (Class III)
DNA binding by OASIS Dimitrov et al. (2016)	AN2
Genetic toxicity Dimitrov et al. (2016)	Negative

§ Ostwald coefficient for O_2_ in Water at 308 K and 1 atm is about 0.028 (Rettich et al., 2000).

## Computed Tomography Perfluorocarbon Emulsion Contrast Agents

In computed tomography, an X-ray beam is rotated circumferentially around the patient while measuring the X-ray transmission at each interval (Hasebroock and Serkova, 2009). Iodinated contrast media are common for computed tomography, particularly for angiography, but also for identifying lesions (Hasebroock and Serkova, 2009). These are often substances like *Lipiodol®* which is derived from poppy seed oil. This medium can be taken up by tumours for cases like hepatocellular carcinoma, where the oil remains longer than in healthy tissue, allowing contrast enhancement on CT images (Rasmussen, 2008). Much initial success for perfluorocarbon contrast enhancement came during studies involving radiopaque brominated perfluorocarbon for radiography, namely PFOB (Long et al., 1982a; Wolf et al., 1994; Hirschl et al., 1996; Riess, 2001; Riess, 2005; Tak and Barraclough, 2018). Perfluorohexylbromide (PFHB) and PFOB, have long been studied as CT contrast agents for viewing areas like the bronchia, gastrointestinal tract, and tumours (Patronas et al., 1983).

As the bromine halogen provides radiopacity, intravenous PFOB emulsified with lecithin at doses between 1 and 3 g. kg^−1^ were previously tested in human studies as a CT contrast agent to image the blood vessels, liver, and spleen (Bruneton et al., 1989). The substance was effective at identifying small metastatic lesions and distinguishing blood vessels from small lesions, compared to non-contrast CT. Tumour lesion enhancement of small metastatic lesions on MRI, CT, and ultrasonography, has been observed after intravenous injection of PFOB emulsions; thought to be attributed to the enhanced permeability and retention effect or macrophage phagocytosis (Mattrey, 1989). Perfluorocarbons have shown utility in diagnostic X-ray radiography for bronchography and alveolography in humans (Long et al., 1982b; Voynikov et al., 1990). CT contrast agent perfluorocarbon emulsions have shown attenuation increases in dogs and pigs of 117 Hounsfield units (HU), 77 HU, and 54 HU in the vasculature (∼13–50 HU normal), spleen (∼45 HU normal), and liver (∼60 HU normal), respectively (Mattrey et al., 1984). These values are very similar to attenuation increases reported with commercial iodinated contrast agents (Amato et al., 2013). It has also been shown as a macrophage-specific CT contrast agent of liver tumours (Voynikov et al., 1990). *Ftoran-RK* has also been reported as an effective contrast agent for X-ray CT attenuation, composed of PFOB and perfluoromethycyclohexylpiperidine (PFMCP), emulsified with poloxamer *Proxanol-268*, with 50–80 nm diameter, and a circulatory half-life around 24 h (Vorob’ev, 2009; Vorob’ev et al., 1993). Additionally, perflubron injection was used as a contrast agent for lymph node CT imaging in human volunteers with only site injection discomfort as a reported side-effect (Hanna et al., 1994).

## Magnetic Resonance Imaging Perfluorocarbon Emulsion Contrast Agents

MRI uses radiofrequency (RF) to excite protons and measure the RF emissions as the protons relax to equilibrium. Inside the bore, the protons align in the direction of the scanner’s main, longitudinal, magnetic field. The protons do not align completely, but precess at a resonant frequency around the direction of the net magnetic field (Katti et al., 2011). Then, a RF pulse, with the same frequency as the precessing protons, is absorbed and displaces the spins into the transverse plane. The proton net magnetization vector relaxes towards the main magnetic field, emitting a free induction decay signal that is detected in the RF receiver coil. The amplitude and phase over a range of emitted RF signals is then correlated to the intensity and location to generate the contrast seen in the MRI image (Westbrook, 2016). The signal has a T_1_ component in the direction of the main magnetic field and T_2_ component transverse to the main magnetic field direction, with the phase lag generating the image contrast. Magnetic resonance contrast agents generally work by reducing the T_1_ or T_2_ relaxation rates of the tissue in the image, with diseased tissue like tumours having a varying amount of contrast agent than other tissue (Geraldes and Laurent, 2009). Positive contrast agents result in a reduction in T_1_ relaxation rate to create signal hyperintensity, while negative contrast agents alter the T_2_ relaxation rate and generate signal hypointensity (Laurent et al., 2009). Perfluorocarbon contrast agents operate through a different mechanism, reducing the local proton density signal to act as a negative contrast agent.

Molecular imaging is a technique to image biomarkers indicative of a disease, by using ligand-mediated agents to target specific cell receptors; for instance to target molecules highly or exclusively expressed in atherosclerotic plaques, ischemic tissues, and tumours (Krafft and Riess, 2021). The liquid perfluorocarbon emulsions have also been studied as a molecular imaging probe. The emulsions can be conjugated with molecular markers specific to certain cell types, including antibodies, peptides, and oligosaccharides; and the accumulation can be visualized with ^19^F-MRI (Krafft and Riess, 2021). Other contrast agents like gadolinium chelates, iron oxides, hyperpolarized ions, or fluorodeoxyglucose (^18^F-FDG), can be incorporated into the emulsions to allow contrast enhancement with, ^19^F-MRI, ^1^H-MRI, or PET (Wolber et al., 1999; Fabiilli et al., 2013; Amir et al., 2017; Krafft and Riess, 2021). The colloids can also be loaded with drugs and act as theranostics, to both treat the disease and monitor biodistribution (Krafft and Riess, 2021). ^19^F-MRI cell tracking with intravenous perfluorocarbon emulsions has been used in humans in a phase I clinical trial for tracking dendritic cell vaccine immunotherapy during treatment of late-stage colorectal cancer (Ahrens et al., 2014), benefiting from being implemented in previous clinical studies for oxygenation after brain trauma and stroke (Darçot et al., 2020).

*Imagent GI* was a previously FDA-approved unemulsified PFOB oral negative contrast agent for ^1^H-MRI that has since been discontinued (Brown et al., 1991; Bisset et al., 1996; Geraldes and Laurent, 2009). PFOB molecules contain no hydrogen atoms and create a proton density weighted image, effectively darkening the bowel loop, allowing better delineation of blood vessels and abdominal organs like the spleen, liver, kidneys, and pancreas (Mattrey et al., 1988; Mattrey, 1989; André et al., 1990; Mattrey et al., 1991). Also, the use of ^19^F-MRI with gaseous unemulsified perfluorocarbon as a contrast agent for respiratory disease has completed many early phase clinical trials (Couch et al., 2013; Halaweish et al., 2013; Goralski et al., 2020; Krafft, 2021). Perfluoropropane (PFP) and ^19^F-MRI has shown in human trials the ability to distinguish healthy and diseased lungs from patients with COPD, cystic fibrosis, asthma, and emphysema. (Halaweish et al., 2013; Couch et al., 2019; Gutberlet et al., 2018) ^19^F-MRI of the lungs can provide functional imaging and generally uses a mixture of about 21% oxygen and 79% PFP or sulfur hexafluoride (SF_6_) (Couch et al., 2013). Healthy volunteers show a homogenous distribution of PFP throughout the lungs, while patients with diseased lungs show incomplete heterogeneous gas distribution. The technique has also shown useful to assess proper lung function from patients that received a lung transplant (Halaweish et al., 2013). The modality would surely be useful in understanding the effects of coronavirus disease 2019 (COVID-19) on lung structure and function, though a specialized MRI receiver coil is required, and to date no studies have reported results; though at least one trial has been initiated (NCT04872309).

## Other Applications

Though, radiological studies have indicated the efficacy, many preclinical formulas have yet to undergo costly toxicological safety studies needed for an investigative new drug application, to permit clinical studies on humans. Much of the literature describing toxicological safety studies of perfluorocarbon emulsions has been for blood oxygenation (Spahn, 1999; Leese et al., 2000; Noveck et al., 2000; Hill et al., 2002; Spiess, 2009; Hill, 2019). *Perftoran*, also known as Perfluorane, is a perfluorocarbon emulsion composed of 10% w:v perfluorodecalin and perfluoromethycyclohexylpiperidine, currently approved as an anti-ischemic and antihypoxant drug in the Russian Federation, Uzbekistan, and Mexico, and was previously approved in many former Soviet states (Khan et al., 2020; Maevsky et al., 2020). *Perftoran* has been shown to induce vasodilation in patients with vascular disease; including patients with limb ischemia, atherosclerosis, diabetes mellitus, oedema after trauma, and oedema post-surgery (Moroz et al., 2007). These emulsions have also shown to improve preterm birth outcomes during gestosis and preeclampsia, which can exhibit acute damage to the peripheral vasculature, platelet damage, vessel constriction, and organ hypoperfusion; in severe cases, leading to acidosis and organ failure. When used in combination with cytoflavin, improved outcomes have been shown in the treatment of moderate preeclampsia, by increasing vascular perfusion and reducing hypoxia, to prolong pregnancy (Kachalina et al., 2007). Moreover, human studies have shown decreased mortality during severe sepsis by oxygenation and improved microcirculation (Yermolenko et al., 2007). *Perftoran* has shown improved outcomes when administered intra-operatively during lobectomy of lung cancer patients with severe respiratory disorders (Kligunenko et al., 2007). The developers have indicated the need and utility for re-establishing large-scale industrial production for the treatment of COVID-19 (Maevsky et al., 2020).

Liquid perfluorocarbons are also used extensively in ophthalmological surgery for applications including: giant retinal tears, vitreoretinopathy, and retinal detachment repairs (Kramer et al., 1995; Mikhail et al., 2017). Also, the perfluorocarbon emulsions are under study as potential focused ultrasound adjuvants due to their enhanced absorption of ultrasonic energy and resulting increasing heat generation (Schad and Hynynen, 2010; Zhang et al., 2011; Kopechek et al., 2013; Phillips et al., 2013; Moyer et al., 2015; Desgranges et al., 2019; Lorton et al., 2020). *Echogen* was a previously FDA approved ultrasonography perfluorocarbon phase-shift emulsion, causing a change from a liquid to gas state when imaged with ultrasonography; composed of C_5_F_12_ and an albumin surfactant (Lin and Pitt, 2013). *Perftoran* is effective as an ultrasonography contrast agent in identifying fluid foci liver lesions and for echocardiography (Vakulenko et al., 2021). *Fluosol* emulsions have been used in early phase clinical trials as an adjuvant to radiotherapy for high-grade gliomas, and late-stage squamous cell carcinomas in the neck and head (Lustig et al., 1989; Evans et al., 1990).

## Clearance, Toxicity, and Side-effects

PFOB has been well-studied as an emulsion contrast agent in humans, particularly due to its fast excretion rate (Burgan et al., 1987; Mattrey, 1989); approximately 3 days at 2.7 g. kg^−1^ (Riess, 2001). Intravenous injection of 1 g. kg^−1^ 0.1–0.2 µm lecithin-PFOB emulsions in 60 patients gave no detectable toxicity (Mattrey, 1989). Oral administration of unemulsified gastrointestinal PFOB contrast agent at 2–12 ml. kg^−1^ doses resulted in no toxic symptoms within 3 days in 60 human subjects, with almost all PFOB eliminated within 24 h (Long et al., 1972b). The contrast agent pharmacokinetics can be quantified with MRI, CT, positron emission tomography, gamma counting, high-performance liquid chromatography, and elemental analysis (Pierre and Allen, 2017). Lecithin-PFOB pharmacokinetic studies indicated that intravenous emulsions are opsonized to a large extent by Kupffer cells and splenocytes, resulting in large deposits of PFOB in the liver and spleen within a few minutes of injection (Riess, 2001; Blanco et al., 2015). Here, the emulsions are degraded, then unmetabolized PFOB re-enters the blood stream, binds to plasma lipids, accumulates in the lungs, before being expelled by respiration (Spahn, 1999).

Intravenous lecithin-PFOB at doses between 1 and 3 g. kg^−1^ were previously tested in humans as a CT contrast agent to image the blood vessels, liver, and spleen (Bruneton et al., 1989). The toxicity was assessed with laboratory tests 2 days before and 7 days following using blood samples, electrolytes, liver function, renal function, proteins, and endocrine factors. The side-effects were mainly asymptomatic and included splenomegaly, and abnormal gamma glutamyl transferase, alkaline phosphatase, and blood platelet levels. Slight lower back pain was observed in some patients thought perhaps to result from venous constriction. These symptoms and influenza-like symptoms are typical of perfluorocarbon emulsions, also seen in liposomal parenteral nutrition formulas, and generally all substances with adsorbent surfaces (Vorob’ev, 2009). The side-effects have also been linked with a size-dependence, as smaller emulsions are less detectable by the macrophage phagocytosis system (MPS) (Spahn, 1999). A concentration dependence of side-effects has also been linked to the increased release of cytokines that can result in flushing and fever (Flaim, 1994). Early emulsion formulas incorporated *Proxonol-F68* emulsifier, later replaced by phospholipids or *Proxonol-268*, which were designed to avoid the immune system and resulted in improved circulatory half-lives, reduced cytokine response, and reduced side-effects (Vorob’ev, 2009). Though, not reported to have caused adverse health effects, some long-term accumulation has been suspected from CT imaging of patients whom were administered PFOB for liquid ventilation therapy during severe respiratory distress syndrome (Hagerty et al., 2008; Servaes and Epelman, 2009; Tak and Barraclough, 2018).

The primary constituent in perfluorocarbon contrast agents are a class of chemicals known as perfluoroalkyl substances (PFAS). The adverse reports are generally associated with prolonged environmental exposures. Certain PFAS molecules have very long half-lives of 3.5–8 years and have been indicated in many potential adverse health effects (Olsen et al., 2007; Cardenas et al., 2018). The prolonged exposure to PFAS typically occur through contaminated water and food (Cardenas et al., 2018; Fraser et al., 2012; D’eon and Mabury, 2011). There have been clinical trials and cohort studies of adverse health associated with elevated levels of perfluorooctane sulfonate (PFOS) and perfluorooctanoic acid (PFOA) in blood serum, particularly in pregnant women (Granum et al., 2013; Cardenas et al., 2018; Wikström et al., 2019).

## Path to Clinical Translation

For FDA investigational new drug applications (iNDA), products must show efficacy and safety through a series of *in vitro* and *in vivo* tests. A comprehensive overview of the toxicological testing needed prior to iNDA are given by Andrade et al. (2016) Toxicity studies for contrast agents are generally performed after sufficient image enhancement has been verified, pharmacokinetics are known, and elimination routes have been observed (Pierre and Allen, 2017). The studies are aimed at determining the toxic effects on animals so that the effects can be monitored in human studies and also to determine a limit for no observable adverse effects at higher limits than the desired dose, to create a factor of safety in human studies (Pierre and Allen, 2017). Toxicity studies for MRI contrast agents include local site toxicity, allergies and immunogenicity, genotoxicity, and blood compatibility (Pierre and Allen, 2017).

*In vitro* test kits offer an affordable alternative to some animal testing and include assays for fetal-embryonic development (EMA/CHMP/ICH/544278/1998, 2020), gene toxicology assessment, macrophage and neutrophil function assays (CHMP/167235/2004, 2006), among others. *In vitro* enzymatic screening, like liver microsomal preparations, are used in most pharmaceuticals to assess metabolism of the substance based on the clearance route (Pierre and Allen, 2017). Perfluorocarbon emulsion contrast agents have been shown to accumulate in the liver and spleen, before being cleared through the lungs. Illustrating safety to these organs would certainly be necessary for translation. Cytochrome P450 enzymes metabolize the majority of drugs in the liver and assay testing the drug reaction can limit adverse effects in patients and establish half-maximal inhibitory concentration (IC_50_) values (Lynch and Price, 2007). Other *in vitro* test kits include human colon adenocarcinoma cells (Caco-2) for intestinal permeability, plasma protein binding with ultrafiltration, the Ames test for mutations, micronucleus assay for chromosome damage, and high-throughput screening of hERG channel inhibition for cardiovascular safety effects (Andrade et al., 2016).

Clinical trials for pharmaceutical development generally consist of early phase clinical studies on a small patient cohort to determine pharmacokinetics, dose, efficacy and assess side-effects (Lipsky and Sharp, 2001). Subsequent late-phase clinical trials are randomized controlled trials on larger groups to further test efficacy, adverse reactions, and long-term safety effects; including post-market trials for rare adverse events (Lipsky and Sharp, 2001). The quickest route for clinical translation of a new perfluorocarbon emulsion contrast agent formula might be using previously approved perfluorocarbon and emulsifier components in the formula, or using an off-label commercial perfluorocarbon emulsion formula. During the COVID-19 pandemic, many medical products have received streamlined clinical testing for the treatment, prevention, and diagnosis of COVID-19 (Avdeev et al., 2019). *Remdesivir*, for instance, was repurposed from an Ebola virus therapeutic to treat coronavirus disease after being streamlined in the United States, in only 4 months, from new drug application (NDA) submission to emergency use authorization (EUA) (Avdeev et al., 2019). Gaseous perfluorocarbons with ^19^F-MRI have previously been tested in early phase clinical trials for respiratory complications (Couch et al., 2013; Halaweish et al., 2013; Goralski et al., 2020; Krafft, 2021) and has great potential for assessing effects of coronavirus disease on pulmonary structure and function; recently being initiated in early phase clinical trials for assessing effects on lungs, vasculature, and the heart from patients with COVID-19 (NCT04872309). Additionally, potential exists with perfluorocarbon antihypoxants and blood substitutes as an off-label COVID-19 therapeutic, having previous approval for alternative therapies from the Russian Ministry of Health and FDA. The reports from studies with antihypoxant perfluorocarbon emulsions (Yermolenko et al., 2007; Moroz et al., 2007; Kligunenko et al., 2007; Kachalina et al., 2007; EU/3/20/2383, 2021; EU/3/20/2361, 2021) suggest significant benefit for treating complications associated with severe COVID-19 (Avdeev et al., 2019; Maevsky et al., 2020).

## Conclusion

In this mini review, the physiochemical properties, radiological imaging applications, and previous clinical studies with perfluorocarbon emulsion contrast agents have been discussed. Perfluorocarbons provide useful contrast on CT and MRI and current research with theranostics, molecular imaging, and ^19^F-MRI have great potential for future commercial medical products. Many alternative therapies, including blood substitutes and antihypoxants, have been developed using similar formulas to perfluorocarbon emulsion contrast agents. Studies with these alternative therapies have established large amounts of preclinical and clinical data pertaining to biodistribution, clearance, and safety studies, applicable to these imaging agents. Utilizing results from these previous clinical studies and implementing an off-label approach could reduce the complexity to initiate clinical testing. Beyond radiological contrast agents, antihypoxant perfluorocarbon emulsions and diagnostic pulmonary ^19^F-MRI have potential for streamlined clinical translation for the treatment and diagnosis of COVID-19.
